# Cure of chronic hepatitis C virus infection after DAA treatment only partially restores the functional capacity of exhausted T cell subsets: a systematic review

**DOI:** 10.3389/fimmu.2025.1546915

**Published:** 2025-09-01

**Authors:** Ása Didriksen Apol, Christina Sølund, Caroline Vinten, Alexander P. Underwood, Jens Bukh, Nina Weis

**Affiliations:** ^1^ Department of Infectious Diseases, Copenhagen University Hospital, Hvidovre, Denmark; ^2^ Copenhagen Hepatitis C Program (CO-HEP), Department of Infectious Diseases, Copenhagen University Hospital Hvidovre, Hvidovre, Denmark; ^3^ Department of Immunology and Microbiology, Faculty of Health and Medical Sciences, University of Copenhagen, Copenhagen, Denmark; ^4^ Department of Clinical Medicine, Faculty of Health and Medical Sciences, University of Copenhagen, Copenhagen, Denmark

**Keywords:** exhaustion, chronic hepatitis C, direct acting antivirals, hepatitis C virus, T cell, NK cell

## Abstract

**Introduction:**

Chronic hepatitis C virus (HCV) infection drives T cells into a dysfunctional state due to persistent antigen exposure. This state persists despite viral clearance with previously used interferon-based treatments. Treatment exclusively with direct-acting antivirals (DAAs), introduced in 2014, has revolutionised HCV treatment with cure rates exceeding 95%. This systematic review investigates whether HCV cure by DAA treatment restores the functional capacity of different exhausted T cell subsets.

**Methods:**

We systematically searched the databases PubMed and Embase on June 26^th^, 2024, for studies assessing T cell exhaustion post-cure by DAA treatment. Eligibility criteria included interferon-free DAA treatment of adult patients with chronic HCV infection, with no co-infection with hepatitis B virus (HBV) or human immunodeficiency virus (HIV). Studies meeting all inclusion and no exclusion criteria were eligible for full-text screening, and only studies presenting original data were included.

**Results:**

The search identified a total of 448 articles, with 35 articles eligible for full-text screening. Among these, 26 met the inclusion criteria and were included in this systematic review. A total of 919 individuals with chronic HCV infection were included. Following HCV cure, most T cell subsets showed only partial restoration of function. Notably, advanced stages of fibrosis were associated with sustained exhaustion across multiple T cell subsets.

**Conclusions:**

This systematic review found that exhausted T cell subsets are only partially restored after HCV cure by DAA treatment. Severe fibrosis, which can be considered a proxy for the duration of infection, appears to impede the reversal of the immune dysfunction. Further studies are warranted to better understand the influence of potential confounders such as age, sex, fibrosis stage, and duration of infection on the restoration of immune function to gain essential insights for future research.

**Systematic review registration:**

https://www.crd.york.ac.uk/prospero/, identifier CRD42024540474.

## 1 Introduction

Hepatitis C virus (HCV) infection affects around 50 million people globally, contributing to an estimated 242,000 deaths annually according to the World Health Organisation (WHO) (1). Following acute infection, approximately 20-30% will spontaneously clear the virus, while the remainder will develop chronic infection, defined as the presence of HCV RNA in the blood >6 months. The disease is considered a major health risk, with 10-20% of the patients with chronic HCV infection progressing to conditions such as liver inflammation, fibrosis, cirrhosis, and hepatocellular carcinoma (HCC) (2).

HCV displays extensive genetic heterogeneity, and has been classified into multiple major genotypes, with important implications for diagnosis and treatment (3). Previously, patients with chronic HCV infection were treated with a combination of injections with pegylated interferon alpha and ribavirin tablets. The treatment had to be given for 24–48 weeks with a cure rate of about 40-80%, depending on the viral genotype, and many patients experienced severe side effects (4). The introduction of oral combination treatment exclusively with direct-acting antivirals (DAAs) in 2014 revolutionised treatment outcomes, achieving successful cure rates >95% (4). DAAs target essential components of the HCV replication cycle, including the HCV protease, the HCV NS5A protein involved in HCV replication and packaging of virions, and the HCV polymerase, which has the key function of replicating the HCV’s viral RNA (4). Individuals who have cleared HCV, either spontaneously or by DAA treatment, remain susceptible to reinfection, and the rates of reinfection are the highest among vulnerable populations, such as persons who inject drugs (PWIDs) intravenously (2, 5, 6). Despite the remarkable success of DAA treatment in curing HCV infection, barriers such as non-adherence and ongoing substance use continue to hinder treatment initiation in vulnerable populations (7).

Despite ongoing efforts, no attempts to develop a vaccine against HCV have been successful, emphasising the need for a better understanding of both successful and unsuccessful immune responses in HCV infection (8). Recognising this critical gap, the WHO recently highlighted HCV vaccine research as a global health priority (9), underscoring the importance of expanding our knowledge of HCV-related host immunity.

**Figure 1:**
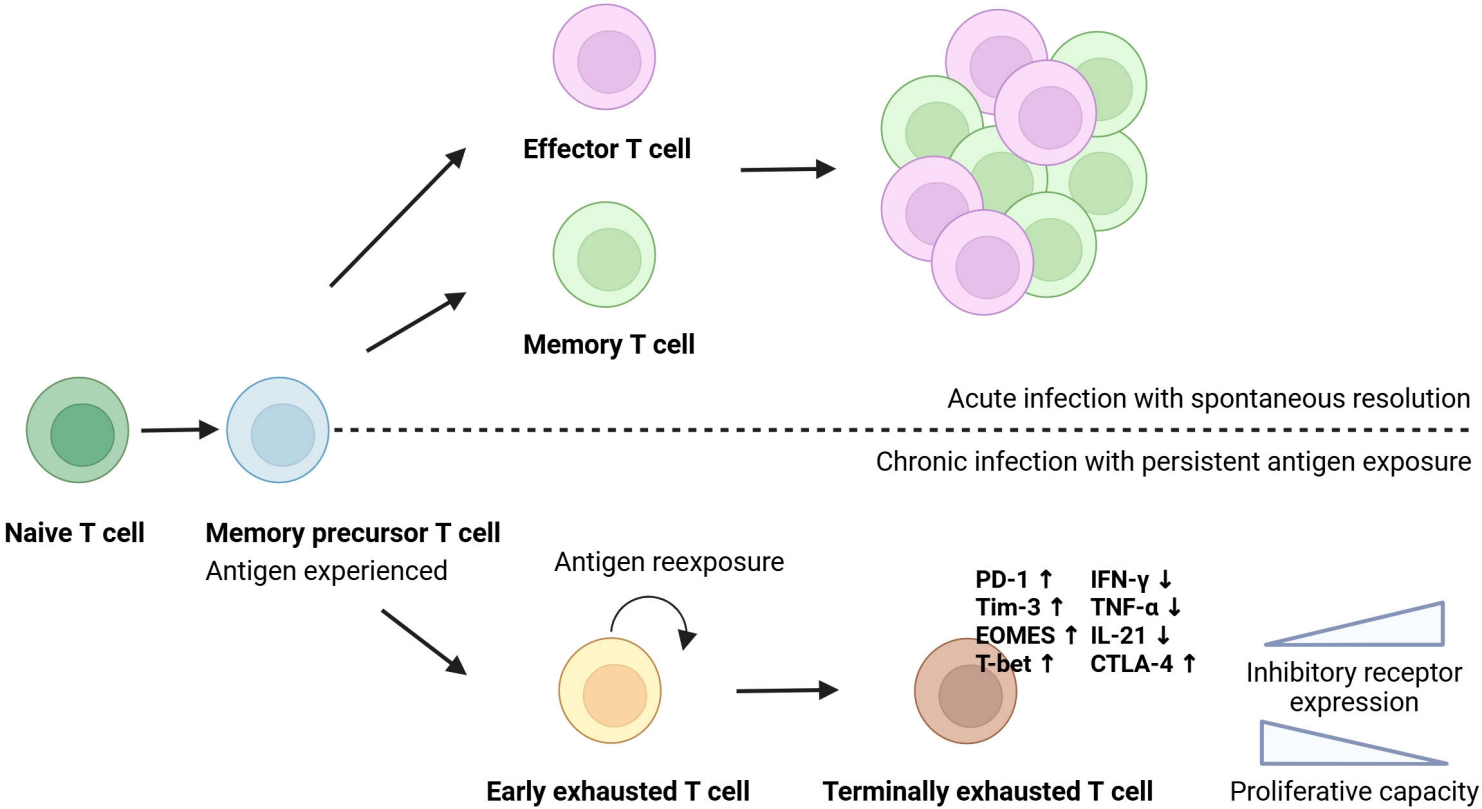
T cell exhaustion. T cells originate from a common precursor and differentiate upon antigen encounter. In acute infection with spontaneous viral clearance, naïve T cells differentiate into functional effector and long-lived memory cells. During chronic viral infection, continuous antigen stimulation drives the cells to a terminally exhausted state, characterised by the expression of inhibitory molecules, reduced cytokine production, impaired proliferative capacity, and distinct transcriptional changes. Created in Biorender.

Robust and multifunctional immune responses are critical in the host’s control of HCV (10). During acute HCV infection, HCV-specific CD (cluster of differentiation)8^+^ T cells are characterised by the expression of activation-associated molecules, such as CD39, along with elevated levels of the transcription factor T-bet, and low levels of cytokine production (11). After elimination of HCV, the phenotype of the CD8^+^ cells transitions towards a memory-like phenotype characterised by high expression of CD127 (interleukin (IL)-7 receptor) (11, 12), which is believed to increase the likelihood of viral resolution in reinfected patients (11). In chronic HCV infection, continuous viral antigen stimulation is thought to drive T cells towards a state of exhaustion, marked by a critical impairment of T cell effector functions (12, 13). It is described as a gradual process where some functions are lost before others, starting with a downregulation of IL-2 production by both CD4^+^ and CD8^+^ T cells. This is followed by a further decrease in the production of other antiviral cytokines such as tumour necrosis factor (TNF-α), interferon-γ (IFN-γ) and beta-chemokines by CD8^+^ cells (10). The exhausted T cells upregulate multiple co-inhibitory molecules, including PD-1 (programmed cell death-1), Tim-3 (T cell immunoglobulin and mucin-domain containing-3), LAG-3 (lymphocyte-activation gene 3), and 2B4 (CD244), and CD8^+^ T cells show an increased expression of the transcription factors EOMES (similar to **Figure 1**) (Eomesodermin) and TOX (Thymocyte selection-associated high mobility group box protein), while displaying low expression levels of CD127 (11). In the final exhaustion stage, the T cells lose their ability to proliferate (14) (**Figure 1**).

Several studies have investigated whether clearance of HCV with DAA treatment leads to reinvigoration of the exhausted T cells, and therefore, this systematic review seeks to summarise the existing research on this topic. Specifically, it will focus on different subsets of T cells: CD8^+^ T cells, CD4^+^ T cells, and unconventional types like mucosal-associated invariant T (MAIT) cells and gamma-delta T cells (γδ T cells), as well as natural killer cells (NK cells). This investigative systematic review will mainly evaluate the function of these immune cell subsets through changes in innate immunity receptors, co-inhibitory molecules, metabolic pathways, cytokine function, and epigenetic control.

## 2 Methods

### 2.1 Search strategy and eligibility

This systematic review follows the Preferred Reporting Items for Systematic Review and Meta-analyses (PRISMA) checklist (2020) (15). The literature search for this review followed a search protocol designed for this study. The literature search was conducted using PubMed and Embase on June 26^th^, 2024. The search was composed using the PICO (Population, Intervention, Comparison, Outcome) method. The search question for this study was: “Is the function of exhausted T cell subsets and NK cells regained after cure by Direct Acting Antivirals in chronic hepatitis C patients?” (**Supplementary Table 1**). The records were screened by two reviewers (ÁDA.; CV). The study was registered in PROSPERO (https://www.crd.york.ac.uk/prospero/) accessed on May 15^th^, 2024, with ID number CRD42024540474.

Only studies presenting original data were included in this review. The search had no date limits, but since DAAs were first introduced in 2011, initially given with interferon, no studies before that were eligible to be included. One article in Russian was discarded. We used Rayyan, a web tool for systematic reviews (16), for abstract screening and to remove doublets, and subsequently conducted full-text screening on all included articles.

For the purposes of this review, ribavirin was considered as a DAA to reflect its inclusion in combination therapies for the treatment of chronic HCV infection.

### 2.2 Inclusion and exclusion criteria

The inclusion criteria of this study were: 1) Studies on adult patients ≥18 years of age with chronic HCV infection defined as HCV RNA positive for >6 months, 2) Cure of chronic HCV infection by treatment with interferon-free DAA regimens defined as sustained virological response (SVR): HCV RNA negative 12 weeks after End Of Treatment (EOT) with DAAs, 3) Outcome: Function of T cell subsets and NK cells. The exclusion criteria of this study were: 1) Co-infection with human immunodeficiency virus (HIV) or hepatitis B virus (HBV), 2) Missing samples either before or after DAA treatment, 3) Review articles, case reports, and animal studies.

## 3 Results

### 3.1 Search results

**Figure 2:**
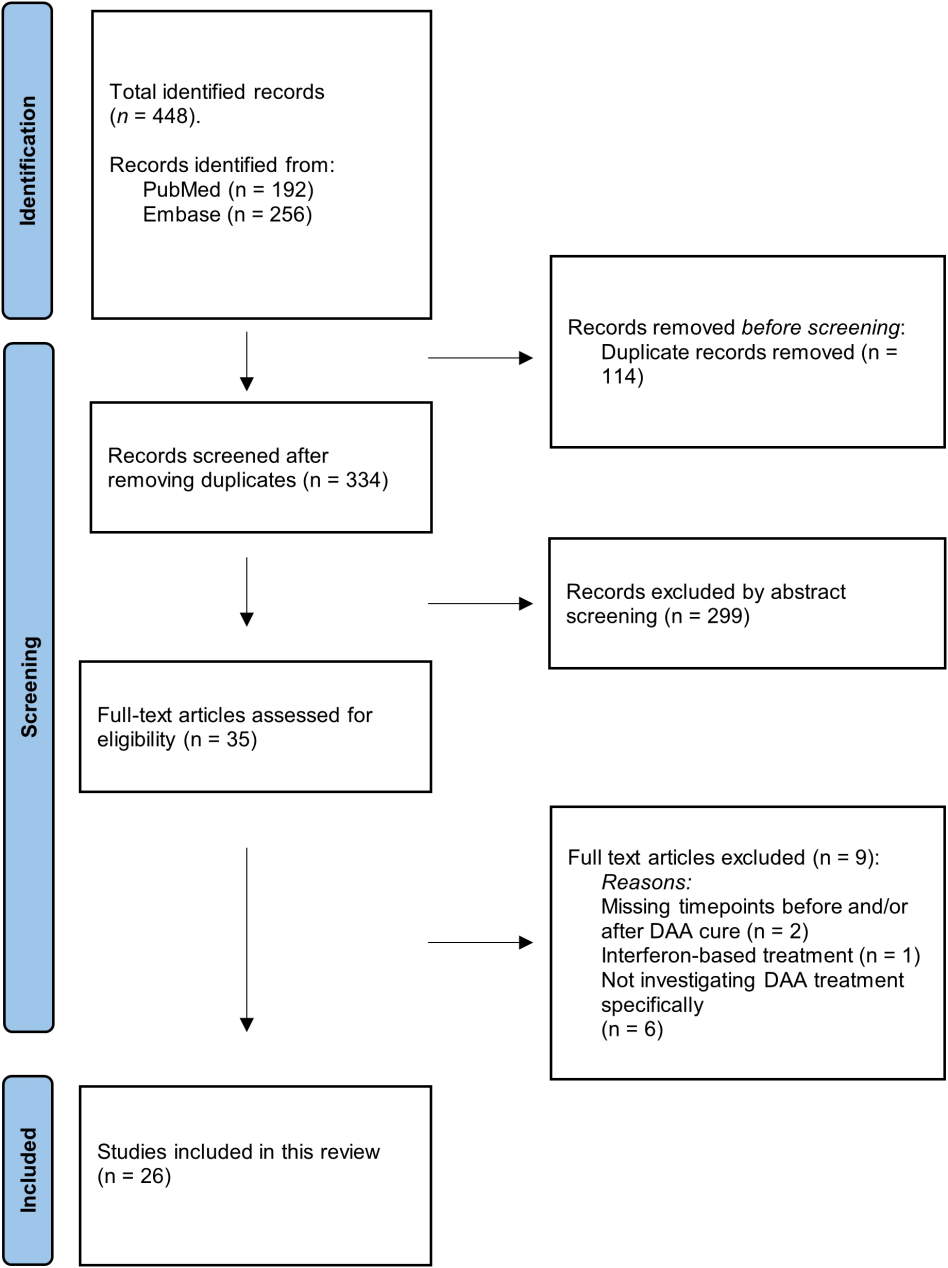
PRISMA flowchart. Illustration of the screening process. The number of records identified, included, and excluded is shown, as well as the reason for exclusion of records.

This systematic review identified a total of 448 articles including 192 articles from PubMed and 256 articles from Embase. When duplicates were removed a total of 334 articles remained. 299 were excluded by abstract screening. The last 35 met the inclusion criteria. Nine articles were excluded after the full-text screening. 26 articles were found eligible and were included in this systematic review (**Figure 2**).

### 3.2 Study design, characteristics, and participants

The study populations of the included studies ranged from six to 108 patients, and a total of 919 individuals with chronic HCV infection were included in this review. Some studies included a control group of healthy subjects (HCV-negative individuals). An overview of the included studies with the DAA treatment regimen, the number of patients, control group, length of the follow-up period, and results is presented in **Table 1**. All the included studies are non-randomised studies of intervention of the subtype “controlled before and after”.

**Table 1:** Overview of the 26 studies included in the systematic review.

First author (reference number) (Publication year)	Direct acting antiviral (DAA) treatment	Study population with HCV (number of individuals)	Control group	Follow-up visits	Cell subset	Main results
Aregay et al. (19) (2019)	SOF or SOF + LDV or SOF + SMV or SOF + DCV or SOF, VEL, and VOX or OBV, PTV, and RTV + DSV or GLE, PIB or EBR + GZR ± RBV (12 weeks)	40	7 healthy subjects	Baseline, EOT, and 24 weeks post EOT	HCV-specific CD8^+^ T cells	Unchanged PD-1, Tim-3, LAG-3, and CD5 expression on HCV-specific T cells post-DAA treatment. Impaired production of IFN-γ, MIP-1β and mitochondrial dysfunction and metabolic deregulation. Decreased HLA-DR^+^CD38^+^ expression on HCV-specific CD8^+^ T cells. Maintained memory-like TCF-1^+^CD127^+^PD-1^+^ HCV-specific T cells.
Barili et al. (29) (2020)	OBV, PTV and RTV + DSV or EBR + GZR or SOF + LDV (12 weeks)	20	28 healthy subjects 26 spontaneously resolved aHCV	Baseline, and EOT	HCV-specific CD8^+^ T cells	Decreased glucose import in HCV-specific CD8^+^ T cells post-DAA. Decrease in mitochondrial depolarisation. p53 was increased compared to the control group. Inhibitors of p53 and histone methylation increased the frequency of both single-positive and double-positive IFN-γ/TNF-α producing CD8^+^ T cells.
Burchill et al. (35) (2015)	DCV, ASV and BMS-791325 (treatment length not specified)	19	None	Baseline, 24 weeks post EOT	Global CD8^+^ and CD4^+^ T cells HCV-specific T cells	Increased frequency of CD4^+^ T cells, decreased frequency of TIGIT expressed on CD4^+^ T cells and CD8^+^ T cells. Increased frequency of T_em_ cells in CD4^+^ and CD8^+^ T cells. Relative decrease in PD-1 expression on HCV-specific T cells post-DAA treatment
Farcomeni et al. (36) (2021)	Not specified	10	10 HCV/HIV co-infected subjects	Baseline, 1 week post treatment initiation (TI), 2 weeks post TI, EOT, 36 weeks post EOT	Global CD8^+^ and CD4^+^ T cells	Increase in CD4^+^ and CD8^+^ T cells post-DAA treatment. Decrease in activation markers CD69^+^ and CD38^+^ in CD4^+^ T cells along with PD-1.
Ghosh et al. (42) (2019)	Group 1: 28 treated with 4 weeks of SOF, LDV, and GS-9451/GS-9699 (4 weeks) Group 2: 5 treated with SOF, VEL and VOX (12 weeks)	28 + 5	15 healthy subjects	Group 1: Baseline, and EOT Group 2: Baseline, 12 weeks post EOT	Global γδ T cells	Vγ9Vδ2 T cells still had phenotypic and functional changes post-DAA treatment with reduced ability to proliferate the CD56^+^ subset.
Hengst et al. (44) (2016)	SOF + RBV (24 weeks)	26	12 healthy subjects	Baseline, 4 weeks post TI, 12 weeks post TI, EOT, 12 weeks post EOT, and 48 weeks post EOT	Global MAIT cells	Low frequency of MAIT cells in HCV infected patients. Increased frequencies of granzyme B, HLA-DR, PD-1, and CD69 and lower expression of CD127 compared to healthy subjects. HLA-DR and CD69 expression partially normalised post-DAA treatment, granzyme B and PD-1 remained elevated and CD127 decreased. Low levels of IFN-γ and TNF-α upon stimulation in HCV infected patients compared to healthy subjects. No significant recovery in MR1-dependent MAIT cell responses post-DAA treatment. T-bet was slightly increased in HCV infected patients and normalised post-DAA treatment.
Hensel et al. (21) (2021)	Not specified	15	8 spontaneous resolvers	Before and after cure (not specified)	HCV-specific CD8^+^ T cells	CD8^+^ T cells both have characteristics of exhaustion (TOX and Eomes expression) and memory (TCF-1 and Bcl-2) post-DAA treatment.
Huang et al. (38) (2022)	Not specified	108	43 healthy subjects	Baseline, and EOT	Global CD8^+^ T cells	CD38^+^HLA-DR^+^CD8^+^T cells primarily belong to the effector memory subset and are many in HCV patients. They have high expression of T-bet and Eomes. CD38^+^HLA-DR^+^CD8^+^T cells displayed high levels of IFN-γ, IL-15, and IL-18 and lower levels of IL-2. The amount of CD38^+^HLA-DR^+^CD8^+^ T decreased post-DAA treatment.
Khanam et al. (40) (2020)	SOF + LDV (12 weeks)	20	15 healthy subjects	Baseline, and 12 weeks post EOT	Global and HCV-specific CD4^+^ T cells	Global CD4^+^CXCR5^+^T_FH_ frequency did not change post-DAA treatment. CD4^+^CXCR5^+^T_FH_ subsets effector memory cells, central memory cells, and terminally differentiated effector memory cells increased. Naïve cells remained the same. Th1 and Th17 on T_FH_ increased and Th2 decreased. Unchanged PD-1 expression on both global and HCV-specific CD4^+^CXCR5^+^T_FH_ cells. HCV-specific cytokines IL-21, IL-17A, IL-22, IFN-γ and TNF-α increased in CD4^++^CXCR5^+^T_FH_ cells post-DAA treatment.
Kochanowicz et al. (45) (2023)	Not specified	97	None	Baseline, 6 months post EOT	Global double-positive CD4^+^CD8^+^ T cells	The percentage of double-positive (DP) T-cells expressing either PD-1 or Tim-3 did not change significantly following treatment. No statistically significant differences in the percentages of DP T-cells with neither PD-1 nor Tim-3 expression. The percentage of DP T-cells co-expressing PD-1 and Tim-3 significantly decreased after treatment.
Llorens-Revull et al. (28) (2021)	SOF + LDV ± RBV or SOF + SMV or EBR + GZR + RBV or OBV, PTV and RTV + DSV + RBV (12 weeks)	27	None	Baseline, 4 weeks post TI, EOT, 12 weeks post EOT	Global CD4^+^ T cells HCV-specific CD8^+^ and CD4^+^ T cells	The surface expression of PD-1, Tim-3, and LAG-3 significantly decreased in CD4^+^ T cells post-DAA treatment when adjusted for clinical parameters like fibrosis stage. Cytokine production by HCV-specific CD4+ and CD8+ T cells remained impaired post-DAA treatment; however, increase in IL-2 in HCV-specific CD8^+^ T cells post-DAA treatment stimulated with core-antigen. Non-cirrhotic patients had a significant improvement in the proliferative capacity of HCV-specific CD4^+^ and CD8^+^ T cells.
Maretti-Mira et al. (31) (2023)	1 with SOF + LDV 1 with SOF + LDV + RBV 1 with OBV, PTV, and RTV + RBV (12 weeks)	11 (4 treatment naïve chronic HCV, 4 spontaneous HCV resolvers, 3 HCV patients during DAA treatment	3 healthy subjects	Baseline, week 2 post TI, 4 weeks post TI, 8 weeks post TI, 12 weeks post EOT	HCV-specific CD8^+^ T cells	Increase in the proportion of CD8+ naïve T cells in successfully treated subjects. No changes in A1 HCV-specific proliferating or TCM phenotypes rates during DAA treatment. A significant reduction in the proportion of T effector memory cells during DAA treatment and a slight decrease in CD8+ exhausted T cells during treatment. A decrease in the expression of HAVCR2 (Tim-3), Eomes, and LAG3.
Martin et al. (18) (2014)	DBV + FDV ± RBV (16–40 weeks)	51	None	Baseline, 4 weeks post TI, EOT, 12 weeks post EOT, 24 weeks post EOT	HCV-specific CD8^+^ T cells	Increase in HCV-specific CD8^+^ T cells after DAA treatment. Phenotypic changes of HCV-specific CD8^+^ T cells in CD127 and PD-1 expression.
Mele et al. (47) (2021)	Not specified	59	34 healthy subjects	Baseline and EOT	Global NK cells	Increased expression of PD-1, TRAIL, and CD25 in HCV patients was normalised post-DAA treatment.
Orr et al. (34) (2020)	SOF + RBV (24 weeks)	40 (24 who achieved SVR and 16 with relapse)	None	Baseline and EOT	Global CD8^+^ and CD4^+^ T cells	Decrease in total lymphocytes, NK cells, CD4^+^ and CD8^+^ T cells. Increase in neutrophil and CD14^+^ monocytes.
Osuch et al. (24) (2020)	54 treated with SOF + LDV (12 weeks) 22 treated with OBV, PTV and RTV + DSV (12 weeks)	76	18 healthy subjects	Baseline, EOT, and 24 weeks post EOT	Global and HCV-specific CD8^+^ T cells Global CD4^+^ T cells	Decrease in global CD8^+^ T cells expressing Tim-3 and co-expressing Tim-3 and PD-1 post DAA. Increase in global CD8^+^ T cells expressing PD-1. HCV-specific CD8^+^ T cells decreased post-DAA treatment but the expression phenotype of PD−1 and Tim−3 remained unchanged post-DAA. Decrease in the amount of CD4^+^ T cells expressing Tim-3. Increase in the amount of CD4^+^ T cells co-expressing Tim-3 and PD-1. The higher the fibrosis stage the less likely a change in exhaustion markers.
Perpiñán et al. (26) (2020)	SOF + LDV ± RBV or OBV, PTV and RTV + DSV + RBV (12 weeks)	26 cirrhotic patients 9 mild HCV non-cirrhotic patients	10 healthy subjects	Baseline, 4 weeks post TI, EOT, 36 weeks post EOT	HCV-specific CD8^+^ T cells	Increased frequency of CD8^+^ T cells post-DAA treatment. Impaired cytokine production.
Shive et al. (37) (2023)	Not specified	15	70 healthy subjects - 40 young adults* - 30 elderly**	Baseline, 4, 12, 24, 48 weeks post TI	Global CD8^+^ and CD4^+^ T cells	A significant decrease in plasma levels of IP10 after initiation of DAA treatment but no significant decrease in plasma levels of IL-6, sCD14, or sCD163. No significant decrease in plasma levels of Endocab, LBP, after initiation of DAA treatment. Decreased co-expression of CD57+TIGIT+ on CD4 T cells 4 weeks post-DAA treatment initiation and a decrease in co-expression of PD1+CD57+ on CD8 T cells at 4 and 24 weeks post-DAA treatment initiation.
Shrivastava et al. (25) (2016)	SOF + LDV (12 weeks)	14 (previous relapsers after SOF + RBV for 24 weeks)	None	Baseline, and EOT	Global CD8^+^ and CD4^+^ T cells	Decreased PD-1, Tim-3 and PD-1 expression in global CD4^+^ and CD8^+^ T cells post-DAA treatment.
Smits et al. (39) (2020)	Not specified	76	Not specified	Baseline, 2 weeks post TI, EOT, 24 weeks post EOT	HCV-specific CD4^+^ T cells	Increased level of CD4^+^ T cells at week 2 post-DAA treatment initiation, which normalised to baseline level at the rest of the time points. No change in the percentages of cells expressing PD-1, BTLA, and TIGIT. Increased level of follicular T helper cells (Tfh).
Stevenson et al. (23) (2019)	SOF (12 weeks)	40	None	Baseline, 4 weeks post TI, EOT, 12 weeks post EOT	Global and HCV-specific CD8^+^ T cells Global CD4^+^ T cells Global NK cells	Decreased PD-1, HLA-DR, CD28, and Tim-3 expression on global CD8^+^ T cells post-DAA treatment. Decreased PD-1 expression on HCV-specific CD8^+^ T cells post DAA-treatment. No changes in PD-1 expression in global CD4^+^ T cells from baseline to post-DAA treatment, but decrease in TIGIT, HLA-DR, and CD28 expression.
Szereday et al. (33) (2020)	OBV, PTV and RTV + DSV + RBV (12 weeks)	14	None	Baseline, EOT, 12 weeks post EOT, 24 weeks post EOT	Global CD8^+^ and CD4^+^ T cells Global NK cells	Decreased Tim-3 expression on CD4^+^, NK^bright^, and NK T cells. Decreased PD-L1 expression on NK cells and regulatory T cells. Increase in CD8^+^ T cells post-DAA.
Tonnerre et al. (27) (2021)	OBV, PTV and RTV + DSV + RBV (12 weeks)	20	None	Baseline, EOT, 12 weeks post EOT	HCV-specific CD8^+^ T cells	High CD38, HLA-DR, ICOS, and CD69 expression levels in exhausted CD8^+^ T cells (T_EX_ cells), who displayed an effector memory type, high expression of molecules related to T cell exhaustion and a transcription factor profile related to exhaustion. Reduced expression of exhaustion markers CD39, Tim-3, CTLA-4, PD-1, CD95, and TIGIT post-DAA treatment.
Vranjkovic et al. (32) (2019)	OBV, PTV + DSV ± RBV (12 weeks)	18	7 healthy subjects	Baseline, 24 weeks post EOT	Global CD8^+^ T cells	Several CD8^+^ T cell subsets were found to be in a hyper-functional state in fibrosis stage F3-F4 patients, which persisted one year after treatment. A large amount of effector and late effector memory cells in F3-F4 compared to F0-F1.
Wieland et al. (20) (2017)	SOF + LDV (8 or 12 weeks) or SOF + LDV ± RBV (12 weeks) or OBV, PTV and RTV + DSV ± RBV (12 weeks)	29	15 spontaneous resolvers (anti-HCV positive, HCV RNA negative)	Baseline, EOT, 12 weeks post EOT	HCV-specific CD8^+^ T cells	Decrease in the amount of terminally exhausted CD8^+^ T cells of TCF-1-CD127-PPD-1^hi^ subset after DAA treatment. TCF-1^+^CD127^+^PPD-1^+^ positive memory-like CD8^+^ T cells persisted.
Yates et al. (22) (2021)	OBV, PTV and RTV + DSV ± RBV (12 weeks)	6	None	Baseline, 4 weeks post TI, EOT, 12 weeks post EOT, and four patients 60 weeks post EOT	HCV-specific CD8^+^ T cells	Many exhaustion markers are not reversed post-DAA treatment including TOX.

γδ T cell, Gamma-delta T cells; 4-1BB/4-1BBL, Receptor ligand for the immune checkpoint TNF; aHCV, acute HCV; ASV, Asunaprevir; BTLA, B and T lymphocyte attenuator; CD, Cluster of differentiation; cHCV, chronic HCV; CTLA-4, Cytotoxic T-lymphocyte associated protein 4; DAAs, Direct Acting Antivirals; DBV, deleobuvir; DCV, Daclatasvir; DP= Double positive; DSV, dasabuvir; EBV, Elbasvir; Eomes, Eomesodermin; EOT, End of treatment with DAAs; FDV, Faldaprevir; GLE, Glecaprevir; GZR, Grazoprevir; HCV, Hepatitis C virus; HIV, Human immunodeficiency virus; HLA-DR, Humane leukocyte antigen DR; ICOS, Inducible co-stimulator; IFN, Interferon; IL, Interleukin; LAG-3, Lymphocyte-activation gene 3; LDV, Ledipasvir; MAIT, Mucosal-associated invariant T cells; MIP-1β, Macrophage inflammatory protein-1beta; OBV, Ombitasvir; PIB, Pibrentasvir; PD-1, Programmed death-1; PTV, Paritaprevir; RBV, Ribavirin; RTV, Ritonavir; SMV, Simeprevir; SOF, Sofosbuvir; SVR, Sustained virological response; TCF-1, T cell factor 1; TCR, T cell receptor; T_EX_ cells, Exhausted CD8^+^ T cells; TI, treatment initiation; Tim-3, T cell immunoglobulin and mucin-domain containing-3; TIGIT, T cell immunoreceptor with Ig and ITIM domains; TNF, Tumour necrosis factor; TRAF1, The signal transducer of the immune checkpoint receptor ligand 4-1BB/4-1BBL; TRAIL, Tumour necrosis factor-related-apoptosis-inducing ligand; VEL, Velpatasvir; VOX, Voxilaprevir.

* ≤ 50 years old.

** > 65 years old.

### 3.4 Results of individual studies based on T cell subsets

#### 3.4.1 CD8^+^ T cell exhaustion in chronic hepatitis C virus infection before and after DAA treatment

CD8+ T cells play a critical role in the control of HCV infection (17). Due to their direct antiviral functions, HCV-specific CD8^+^ T cells represent a key cell population for studying TCR-driven exhaustion. Several studies have specifically examined HCV-specific CD8^+^ T cell responses before and after HCV cure by DAA treatment. Among the first conducted studies on regeneration of T cell function after DAA treatment is a study by Martin et al. (18) including 51 HCV infected patients. The authors demonstrated a significant increase in the level of HCV-specific CD8^+^ T cells in most of the patients achieving SVR. Interestingly, this was not found in the patients with treatment failure. Improved effector functions, such as T cell proliferation, were observed after treatment, accompanied by a slight decrease in exhaustion marker PD-1 and an increase in CD127 expression (18).

In contrast, Aregay et al. (19) studied 40 HCV infected patients and found that expression of exhaustion markers PD-1, Tim-3, LAG-3 and CD5 on HCV-specific CD8^+^ T cells remained unchanged at EOT and 24 weeks after EOT compared to baseline. Despite viral clearance, cytokine production of IFN-γ and MIP-1β (macrophage inflammatory protein-1beta) by CD8^+^ T cells remained impaired, along with unaltered mitochondrial dysfunction and metabolic degranulation observed before treatment. However, a significantly reduced frequency of CD8^+^ T cells expressing CD38 and CD38 and HLA-DR (human leukocyte antigen DR), and terminally exhausted cells expressing CD39, were found after curative DAA treatment. These findings suggest that, despite a reduction of some exhaustion markers, full CD8^+^ T cell function is not restored after DAA treatment (19).

In another study involving 29 HCV infected patients, Wieland et al. (20) found that both CD127^+^PD-1^+^, CD127^-^PD-1^lo^, and CD127^-^PD-1^hi^ subsets were part of the HCV-specific CD8^+^ T cells pool while antigen was present during active infection. Following DAA cure, only the CD127^+^PD-1^+^ subset was maintained, which is characterised by expression of the anti-apoptotic Bcl-2 and the memory-associated T cell factor 1 (TCF-1). Therefore, the subset both expressed characteristics of exhaustion and T cell memory. The study highlighted a correlation between the CD127^+^PD-1^+^ subset and TCF-1, suggesting a role for TCF-1 in the survival of these cells. Despite displaying memory-like characteristics, CD127^+^PD-1^+^ CD8^+^ T cells differed from those observed after spontaneous HCV clearance, showing higher PD-1 and EOMES expression in those that were cured via DAA treatment, which was indicative of exhaustion. Moreover, impaired cytokine production was found in the memory-like HCV-specific T cells compared with levels in the spontaneously cleared counterparts (20).

Comparable results were found in a study by Hensel et al. (21), who investigated a memory-like HCV-specific CD8^+^ T cell subset (TCF-1^+^CD127^+^PD-1^+^) in 15 HCV infected patients. Flow cytometry of samples pre- and post-DAA treatment revealed characteristics of both a memory-like CD127^high^ cluster (TCF-1 and Bcl-2) and an exhausted CD127^lo^ cluster (TOX1 and EOMES). This indicates partial repolarisation of HCV-specific CD8^+^ T cells from an exhausted phenotype toward a memory-like state after DAA mediated HCV cure, although signs of exhaustion persist. Using single-cell sequencing, the authors identified three subsets of HCV-specific CD8^+^ T cells from HCV infected patients: a memory-like CD127^high^ cluster enriched in genes related to T cell memory (TCF-1, Bcl-2, and CCR7), a CD127^lo^ cluster expressing markers of T cell exhaustion (TOX1, PD-1, CD39, CD38, CD137, and IRF4), and an intermediate CD127^int^ cluster with features from both T cell memory and exhaustion. Post-DAA treatment, the CD127^lo^ cluster was lost, indicating loss of terminally exhausted cells after HCV cure while the memory-like T cell subsets were maintained. However, when they compared the transcriptome of memory-like T cells from HCV infected patients pre- and post-DAA treatment with memory T cells from individuals who spontaneously resolved HCV, they found that both pre- and post-DAA treatment, the memory-like T cell subset was enriched for genes related to T cell exhaustion compared to the memory T cell subset in the spontaneously resolved HCV. This suggests that the molecular program of exhaustion is maintained after DAA-mediated viral clearance, leaving a “molecular scar” even after HCV cure (21).

Similarly, Yates et al. (22) describe an “epigenetic scar” in HCV-specific CD8^+^ T cells by examining chromatin-accessible regions (ChARs) using ATAC-seq in six HCV infected patients before and after DAA treatment. They identified a change of 25,237 ChARs in the samples after DAA treatment, with some exhaustion-related ChARs (e.g., CTLA-4) being lost, while others persisted post-DAA treatment. The ChARs could roughly be divided into two groups: those that remained “scarred” ChARs (enriched for genes related to nuclear factor of activated T cells (NFAT) and hypoxia-inducible factor 1-α (HIF-1α) signalling) and those that were “reversed” (related to translocation of zeta-chain-associated protein kinase 70 (ZAP-70) and PD-1) after treatment. Additionally, genes related to T cell memory were found in gained regions post-treatment. Despite these changes, most ChARs remained scarred, maintaining an exhausted HCV-specific CD8^+^ phenotype even after viral cure. Further, analysis of samples from four patients 60–80 weeks post-DAA treatment was performed, and the ChARs from a late timepoint during treatment were compared to ChARs from the follow-up visit post-treatment. Half of the reversed ChARs were unchanged, while the rest decreased over time. In contrast, 77.1% of the scarred ChARs, including TOX, remained unchanged, indicating that the exhausted phenotype of HCV-specific CD8^+^ T cells persists long-term after cure by DAA treatment (22).

Stevenson et al. (23) included 40 HCV infected patients and observed a decrease in PD-1 expression in the total HCV-specific CD8^+^ T cell pool at post-DAA treatment compared to baseline. In the total CD8^+^ T cell pool, Tim-3 expression also decreased at post-DAA treatment, though this was not observed in the HCV-specific CD8^+^ T cell pool. No differences in neither T cell immunoreceptor with Ig and ITIM domains (TIGIT) expression nor the total number of CD8^+^ T cells was observed. In addition, a decrease in HLA-DR and CD28 was observed from baseline to follow-up, implicating an overall reduction in T cell exhaustion and activation (23).

A larger study involving 76 HCV infected patients by Osuch et al. (24) found a decrease in total CD8^+^ T cells expressing Tim-3 after DAA treatment. The number of global T cells co-expressing PD-1 and Tim-3 decreased, while the number of CD8^+^ T cells expressing only PD-1 increased. The study also highlighted that the more advanced the fibrosis stage, the less likely the treatment was to alter the expression of exhaustion markers. Not surprisingly, HCV-specific CD8^+^ T cells significantly decreased post-DAA treatment but the expression phenotype of PD−1 and Tim−3 remained unchanged (24).

Shrivastava et al. (25) investigated 14 HCV infected patients with a relapse from previous treatment with sofosbuvir plus ribavirin for 24 weeks, and found a decrease in PD-1, Tim-3, and CD57 expression on global CD8^+^ T cells at EOT. The study also examined enhancer functions through PBMC stimulation assays with a panel of HCV peptides, and observed an increase in TNF-α, IFN-γ and IL-2, suggesting enhanced functional capacity at EOT. At EOT, the detection of HCV-specific CD57^+^, PD1^+^ and Tim-3^+^ cells declined, with most cytokine responses produced by cells lacking these exhaustion markers, suggesting that exhausted T cells have indeed lost their functional capacity to produce cytokines (25).

A study by Perpiñán et al. (26) analysed samples of 26 cirrhotic and 9 non-cirrhotic HCV infected patients, and 10 healthy subjects. The frequency of HCV-specific CD8^+^ T cells in the HCV infected patients increased from baseline to follow-up at week 36 post EOT. Both cirrhotic and non-cirrhotic patients exhibited high expression levels of PD-1 on HCV-specific CD8^+^ T cells. Compared with non-cirrhotic patients, cirrhotic patients had high levels of exhaustion markers Tim-3 and CTLA-4 (cytotoxic T-lymphocyte associated protein 4) on HCV-specific CD8^+^ T cells, with persistent co-expression of PD-1/Tim-3 and PD-1/CTLA-4 until the EOT, which only decreased to levels like non-cirrhotic patients by week 36 post EOT. Cytokine production was assessed after *in vitro* stimulation of PBMCs for 12 days, and the authors found that it remained impaired in all HCV infected patients regardless of cirrhosis status, with low frequencies of IFN-γ, TNF-α and MIP-1β production in HCV-specific CD8^+^ T cells in both groups and no increase at follow-up 36 weeks post EOT (26).

Tonnerre et al. (27) conducted a study on 20 HCV infected patients and found a decrease in the number of HCV-specific T_EX_ (exhausted CD8^+^ T cells targeting conserved epitopes) following viral clearance by DAAs. T_EX_ cells were defined by high expression of CD38, HLA-DR, ICOS, and CD69, which are related to a high activation profile. T_EX_ cells also had an effector-memory type (CCR7^lo^, CD45RA^lo,^ and CD127^lo^), high expression of molecules related to T cell exhaustion (PD-1, TIGIT, CD95, BTLA, 2B4, and CD39), and a transcription factor profile related to exhaustion (TCF-1^lo^, EOMES^hi,^ and T-bet^lo^). 12 weeks post EOT, there was a significant reduction in activation markers (complete loss of CD38, HLA-DR, ICOS, CD69 and CD71 expression), with a shift towards a central memory phenotype, indicated by increased CCR7 and CD127 expression and higher TCF-1 frequencies. Although inhibitory molecules like CD39, Tim-3, CTLA-4, PD-1, CD95, and TIGIT were still expressed, their expression rates were significantly reduced, suggesting a transition towards a less exhausted state after DAA treatment, aligning more with memory T cells. Four patients were followed for three years after DAA treatment, and these patients maintained steady expression levels of most molecules, consistent with the levels observed at the early time point after DAA treatment. Specifically, there was a slight increase in CD127 and a slight decrease of Eomes and CD39, but these changes did not affect T cell activation and function (27).

Llorens-Revull et al. (28) examined functional changes of CD8^+^ T cells in 27 HCV infected patients following DAA treatment. Upon stimulation with HCV-antigens, HCV-specific CD8^+^ T cells showed no significant increase in IFN-γ production at 12 weeks post-EOT compared to baseline. However, after stimulation with non-structural (NS)3 helicase or Core peptides, an increase in IFN-γ production was observed in HCV-specific CD8^+^ T cells in the same period. IL-2 production decreased in HCV-specific CD8^+^ T cells in response to NS3 peptide, while IL-2 production increased with Core peptide stimulation. This indicates that cytokine production remains impaired after viral clearance by DAA treatment. Additionally, HCV-specific CD8^+^ T cells displayed enhanced proliferative capacity after NS3 helicase and peptide stimulation, with significant improvement seen only in non-cirrhotic patients when accounting for fibrosis score (28).

In another study, Barili et al. (29) explored the effect of glycolytic and mitochondrial functions, such as reduced cellular respiration and diminished glucose uptake, in HCV-specific CD8^+^ T cells in 20 patients with chronic HCV before and after DAA. Previous research in the LCMV mouse model has linked reduced glycolytic activity to exhaustion in virus-specific CD8^+^ T cells (30). The authors found that glucose import was significantly reduced at EOT in HCV-specific CD8^+^ T cells but did not reach levels found in spontaneous HCV resolvers. Mitochondrial depolarisation declined in HCV-specific CD8^+^ T cells from baseline to EOT in some but not all patients. p53 is a negative regulator of glycolysis and an enhancer of mitochondrial oxidative phosphorylation (OXPHOS) and is involved in the regulation of cell-cycle arrest. Compared to the spontaneous HCV resolvers and healthy controls, the level of p53 was increased in global CD8^+^ T cells in chronically infected HCV patients. Inhibition of p53 and histone methyltransferases before and after DAA treatment increased the frequency of both single-positive and double-positive IFN-γ/TNF-α producing CD8^+^ T cells upon peptide stimulation (29).

A recent study by Maretti-Mira et al. (31) used single-cell RNA sequencing to characterise HCV-specific CD8^+^ T cells in 11 HCV infected patients across different groups: untreated chronic HCV infection (n = 4), spontaneously resolved HCV infection (n = 4), and patients who successfully cleared HCV at EOT (n = 3), compared to healthy subjects (n = 3). The researchers demonstrated that in untreated chronic HCV infected patients, genes associated mainly with cytotoxicity (e.g., cell death induced by granzymes, cytolysis, and cell killing), and with lymphocyte activation (e.g., IL-12 signalling and IFN-γ response), were upregulated. In spontaneous resolvers, the upregulated genes were related to cell viability and cell differentiation and activation, suggesting that after spontaneous resolution, the cytotoxicity of HCV-specific CD8^+^ T cells was reduced. The cytotoxic T cell features in chronic HCV infected patients progressively decreased with DAA treatment and persisted 12 weeks post EOT. Successful DAA treatment led to an increase in the proportion of naïve CD8^+^ T cells, alongside reductions in effector memory CD8^+^ T cells and a slight decrease in exhausted T cells marked by Tim-3, EOMES, and LAG3 expression. However, no changes were seen in HCV-specific proliferating or central memory T cells during treatment or 12 weeks post EOT (31).

Complementing the studies on HCV-specific CD8^+^ T cell responses, several studies mainly focused on global, antigen-non-specific responses. Although these responses are less directly tied to antigen driven T cell exhaustion, they can provide broader insights into immunological changes following antigen clearance. In a study by Vranjkovic et al. (32) on 18 HCV infected patients, the recovery of CD8^+^ T cell function in relation to liver fibrosis was studied. The study compared the effect on global (i.e., not antigen-specific) CD8^+^ T cells after viral clearance between a group of patients with liver fibrosis stage F0-F1 (n = 11) and a group with stage F4 (n = 7). Patients with advanced fibrosis (F4) exhibited hyper-functional CD8^+^ T cell subsets, characterised by elevated perforin production and cytotoxicity, after cure with DAA treatment. The bulk CD8^+^ T cells in the F0–1 group were observed to be more like bulk CD8^+^ T cells in healthy subjects. The HCV infected F4 patient group had less naïve CD8^+^ T cells and a large amount of effector and late effector memory cells compared to both F0–1 patients and healthy subjects. The F4 patients produced more perforin than the F0–1 patients, who also were found to better reduce IFN-γ and CD107a responses after DAA treatment. The hyper-functional state in the F4 patients persisted after cure with DAA treatment and was still found one year post treatment initiation (32).

In another study on 14 HCV infected patients, Szereday et al. (33) reported an increase in global CD3^+^ and CD8^+^ T cells from baseline and follow-up visits 12 and 24 weeks post EOT. The Tim-3 expression on CD8^+^ T cells did not change following treatment with DAAs and no differences were found in PD-1 or PD-L1 expression (33).

Orr et al. (34) investigated a cohort of 40 HCV infected patients, which included a high percentage of individuals with relapse (29%) due to treatment failure. The study found a decrease in total lymphocytes, NK cells, CD4^+,^ and CD8^+^ T cells, along with an increase in neutrophil and CD14^+^ counts during treatment. At EOT, genes associated with T cell activation, *CD80*, *CD160*, *LAG3*, *B and T lymphocyte attenuator* (*BTLA*), and *Inducible co-stimulator* (*ICOS*), showed decreased expression. No association was found between the EOT results and clinical parameters like fibrosis stage and ALT levels. When comparing patients achieving SVR with patients who experienced relapse, the latter had higher NK cell counts post-treatment, as well as higher expression of genes associated with T cell dysfunction post-treatment, including HAVCR2, KLRG1, and CD244 (34).

Burchill et al. (35) conducted a study on 19 HCV infected patients following DAA treatment. The authors found that the number of global CD8^+^ T cells remained unchanged 4 weeks post EOT. Additionally, a higher relative ratio of T-bet to EOMES was observed 4 weeks post-EOT on global CD8+ T cells, which indicates a general switch within the T cell compartment towards a more effector-like state during DAA treatment. Furthermore, the study found downregulation of TIGIT expression on global CD8^+^ subsets. Lastly, in a subgroup of seven HCV infected patients, the authors investigated exhaustion-related markers on HCV-specific cytotoxic T cells at baseline and 24 weeks post-EOT, and found and a relative decrease in PD-1 expression (35).

Farcomeni et al. (36) investigated the expression of activation markers CD69, HLA-DR, CD38 and CD28, PD-1, and naïve/memory markers CD45RA/CD45RO on global CD8^+^ T cells during DAA treatment in 10 HCV infected patients. The percentages of CD8^+^ T cells initially increased from baseline with 30.66% to 42.74% two weeks post-treatment initiation, but slightly declined to 37.60% at 36 weeks post EOT. CD45RA expression on CD8^+^ T cells remained unchanged throughout the treatment period (36).

Shive et al. (37) examined 15 HCV infected patients initiating DAA treatment and observed decreased expression of PD1 and CD57 on global CD8^+^ T cells at four and 24 weeks post-EOT. Further, the authors demonstrated a significant decrease in levels of IFN-gamma-inducible protein 10 (IP10) after DAA treatment initiation. However, there was no decrease in levels of other inflammatory mediators (IL-6, sCD14, and sCD163), nor in endotoxin-core IgG antibody (Endocab IgG) or lipopolysaccharide-binding protein (LBP) after initiation of DAA treatment (37).

Lastly, Huang et al. (38) investigated 108 HCV infected patients, focusing on the subset of the TCR-independent bystander CD38^+^HLA-DR^+^CD8^+^ T cells in patients with chronic HCV infection. While these cells are known to cause liver damage in acute HCV infection, their role in chronic HCV infection is less clear. Compared to healthy subjects, HCV infected patients had higher expression levels of global CD38^+^HLA-DR^+^CD8^+^ T cells. Phenotypic analysis revealed that the majority of the CD38^+^HLA-DR^+^CD8^+^ T cells were of the effector memory subtype (T_EM_; CD45RA^−^CCR7^−^), whereas the majority of CD38^-^HLA-DR^-^CD8^+^ T cells were naïve. The CD38^+^HLA-DR^+^CD8^+^ T cells showed higher expression of CCR3 and CCR5 compared with CD38^-^HLA-DR^-^CD8^+^ T cells, indicating a predominant function as memory T cells. Both T-bet and EOMES, crucial for CD8^+^ cytotoxic T lymphocyte effector and memory function, were elevated in the CD38^+^HLA-DR^+^CD8^+^ T cells. CD38^+^HLA-DR^+^CD8^+^ T cells were also found to display higher IFN-γ and lower IL-2 than their CD38^+^HLA-DR^-^CD8^+^ counterparts among samples from both HCV infected and healthy subjects. Plasma levels of cytokines IL-15 and IL-18 were also elevated. The CD38^+^HLA-DR^+^CD8^+^ T cells were largely non-HCV-specific. The authors found that the proportion CD38^+^HLA-DR^+^CD8^+^ T cells positively correlated with liver fibrosis progression. Lastly, DAA treatment significantly reduced the percentages of CD38^+^HLA-DR^+^CD8^+^ T cells at EOT (38).

In summary, the included studies on CD8^+^ T cells show that DAA treatment leads to selective persistence of HCV-specific memory-like CD8^+^ T cells and decrease of terminally exhausted subsets. Reduction of exhaustion markers is seen in some cases, but seemingly many HCV-specific CD8^+^ T cells retain transcriptional or epigenetic traits of exhaustion, particularly in patients with advanced fibrosis. Global CD8^+^ T cells reveal more variable changes and provide a less direct view of exhaustion reversal.

#### 3.4.2 CD4^+^ T cell exhaustion in chronic hepatitis C virus infection before and after DAA treatment

CD4+ T cells are essential in coordinating antiviral immunity, including supporting cytotoxic T cell function as well as facilitating B cells to generate neutralising antibodies through T follicular helper (Tfh) subsets (17). Fewer studies have specifically investigated CD4^+^ T cell responses during and after DAA, of which most of them focus on global, antigen-non-specific responses. One of the studies investigating HCV-specific CD4^+^ T cell responses was Smits et al. (39), who investigated 76 HCV infected patients and observed that baseline levels of HCV-specific CD4^+^ T cells before treatment generally were low. The frequency of HCV-specific CD4^+^ cells significantly increased during the second week of DAA treatment but subsequently decreased at the following timepoints, returning to baseline levels at 12 weeks post EOT, indicating DAA treatment can reinvigorate the circulating pool of HCV-specific CD4+ during the early stages of treatment. Analysis of inhibitory receptors of HCV-specific CD4^+^ T cells revealed that the expression of BTLA and TIGIT was maintained after DAA treatment 12 weeks post EOT. Interestingly, the expression levels of both CD39 and PD-1 decreased significantly during treatment. The study also noted a shift in the phenotypical characteristics of the HCV-specific CD4^+^ T cells during DAA treatment from Th1 polarisation to Tfh cells, indicating an antigen-specific effect on this subset. Despite the overall decrease in CD4^+^ T cells during treatment, the shift towards the Tfh phenotype persisted (39).

Similarly, Llorens-Revull et al. (28) examined phenotypical and functional changes of CD4^+^ following DAA treatment in the study previously described. They found a non-significant decrease in surface expression of PD-1, Tim-3, and LAG-3 on global CD4^+^ T cells at 12 weeks post EOT compared to baseline. However, after adjusting for clinical parameters such as fibrosis stage, the results became significant. A significant decrease in PD-1 expression was observed in non-cirrhotic, treatment naïve patients under the age of 55, but not in cirrhotic patients. Cytokine production by HCV-specific CD4^+^ T cells showed no significant increase in IFN-γ production after stimulation with HCV-antigens at 12 weeks post EOT compared to baseline. Conversely, IFN-γ production increased after stimulation with NS3 helicase or Core peptides. The percentages of CD4^+^ IL-2^+^ T cells at 12 weeks post-EOT did not significantly change from baseline, even after HCV antigen stimulation. IL-2 production decreased in HCV-specific CD4^+^ T cells in response to NS3 peptide. Similar to CD8^+^ T cells, the proliferative capacity of HCV-specific CD4^+^ improved after NS3 helicase and peptide stimulation, with significant improvement only in non-cirrhotic patients when adjusting for fibrosis score (28).

Khanam et al. (40) studied 20 HCV infected patients and found that levels of CD4^+^CXCR5^+^ Tfh cells, specialised in T cell mediated B cell help and essential for facilitating a protective humoral response, did not change at 12 weeks post-EOT. The authors observed an increase in effector memory cells (CD45RA^-^CCR7^-^), central memory cells (CD45RA^-^CCR7^+^), and terminally differentiated effector memory cells (CD45RA^+^CCR7^-^) among CD4^+^CXCR5^+^Tfh cells, while naïve cell (CD45RA^+^CCR7^+^) numbers remained unchanged 12 weeks post EOT. Investigation of Th1, Th2, and Th17-like subsets of Tfh cells revealed an increase in Th1 and Th17 cells and a decrease in Th2 cells 12 weeks post-EOT. Additionally, a subset of memory Tfh cells named CD4^+^CXCR5^+^CXCR3^+^PD-1^+^ cells increased 12 weeks post-EOT, whereas CD4^+^CXCR5^+^CXCR3^-^PD-1^+^ cells did not increase as much. Similar findings were observed in HCV-specific CD4^+^CXCR5^+^ Tfh cells, suggesting a restoration of both global and HCV-specific Tfh cells and their subtypes. Analysis of exhaustion parameters revealed that CD4^+^CXCR5^+^ Tfh cells displayed high levels of PD-1 that remained elevated at 12 weeks post EOT. ICOS expression on CD4^+^CXCR5^+^ Tfh cells also increased, as did the co-expression of PD-1 and ICOS. Further, the authors found that HCV-specific cytokine secretion of IL-21, IL17A, IL-22, IFN-γ and TNF-α on CD4^+^CXCR5^+^ Tfh cells increased 12 weeks post-EOT using a PBMC stimulation assay (40).

The remaining studies on CD4^+^ T cells focus on global responses, giving a broader view on the systemic immune recovery post-DAA. These data are useful in the understanding of immunological alterations during and after antigen clearance but must be interpreted with caution when discussing HCV-specific exhaustion. One study by Burchill et al. (35) also examined CD4^+^ T cells in the same study mentioned previously. They reported an increase in the total number of lymphocytes and global CD4^+^ T cells in the peripheral blood 4 weeks post-EOT. CD4^+^ T cells exhibited a temporary increase in proliferative capacity upon stimulation through the T cell receptor (TCR). Additionally, a phenotypic shift from central memory (T_cm_) (CD45RA^-^CCR7^+^) to effector memory (T_em_) (CD45RA^-^CCR7^-^) phenotype was discovered in the CD4^+^ T cells. The study also detected a higher relative ratio of T-bet to Eomes, associated with a switch from T_cm_ to T_em,_ in CD4^+^ T cells 4 weeks post-EOT, indicating a shift towards a more effector-like state. Consistent with the findings in CD8^+^ T cells, TIGIT expression on global CD4^+^ T cells was downregulated (35).

Complementing these findings, Farcomeni et al. (36) observed a significant increase in the percentages of global CD4^+^ T cells from baseline to 36 weeks post EOT. Expression of CD69 and CD38 on CD4^+^ T cells declined significantly from baseline to EOT, while HLA-DR levels remained unchanged. CD28 expression on CD4^+^ T cells, essential for immune cell activation and proliferation of naïve and memory T cells, also remained unchanged during the treatment period, as did the expression of CD45RA in CD4^+^ T cells. PD-1 expression on CD4^+^ T cells decreased significantly at 36 weeks post EOT compared to baseline. Additionally, IP-10 levels significantly decreased during treatment, while IFN-γ and IRF7 expression significantly increased (36).

Stevenson et al. (23) demonstrated that total CD3^+^CD4^+^CD25^+^CD127^–^ Treg cell frequency decreased during treatment. The total CD4^+^ T cell pool expressed less PD-1 by EOT compared to baseline, although this reduction was not sustained 12 weeks post EOT. The expression of TIGIT, HLA-DR, and CD28 all decreased by 12 weeks post EOT (23). In contrast, Szereday et al. (33) found no difference in the frequency of global CD4^+^ T cells following DAA treatment 24 weeks post EOT. However, they did observe a decrease in the Tim-3 expression on CD4^+^ T cells from baseline to 24 weeks post EOT, with no significant changes in PD-1 or PD-L1 expression (33). Similarly, Osuch et al. (24) reported a decrease in Tim-3 expression on global CD4^+^ T cells without a corresponding decrease in PD-1 expression six months post EOT. The proportion of CD4^+^ T cells co-expressing PD-1 and Tim-3 increased. This study established a correlation between liver fibrosis stage and CD4, and PD-1 expression: the more advanced fibrosis, the higher the frequency of CD4^+^PD-1^+^ T cells. Conversely, a negative correlation was found between fibrosis score and Tim-3 expression with more advanced fibrosis associated with a lower expression of Tim-3 in CD4^+^ T cells. As noted previously on CD8^+^ T cells, the current study found that the probability of changes in expression of exhaustion markers on CD4^+^ T cells were less likely in patients with advanced liver fibrosis (24).

Shrivastava et al. found a decrease in the expression of PD-1, Tim-3, and CD57 in global CD4^+^ T cells at EOT in a study on 14 relapsers (25). Consistently, Shive et al (37) demonstrated a decreased expression of CD57 and TIGIT on CD4^+^ T cells four weeks after DAA initiation (37).

These studies suggest that DAA treatment leads to partial restoration of CD4^+^ T cells, with variability in exhaustion markers expression, memory differentiation, and cytokine production. HCV-specific CD4^+^ T cells, particularly those with Tfh characteristics, may transiently expand or shift in phenotype during treatment, although expression of markers like PD-1 persisted. Global CD4+ T cell responses overall show reduced exhaustion and activation over time, but the extent of the recovery appears limited in patients with advanced liver fibrosis.

#### 3.4.3 Exhaustion of unconventional types of T cells in chronic hepatitis C virus infection before and after DAA treatment

Gamma delta (γδ) T cells represent an unconventional subset of T cells enriched in solid tissue, such as the liver among the hepatic lymphocyte population (41). These cells express γ and δ chains on their TCR and can be categorised into two main groups: Vδ1 cells, which are predominantly found in the gut and epithelial tissues, and Vδ2 cells, which are typically present in the peripheral blood (41). γδ T cells contribute to a Th1-oriented immune response and are involved in antiviral and anticancer functions (42). Ghosh et al. (42) studied the effect of DAAs on γδ T cells in 33 HCV infected patients and found similar numbers of these cells in HCV infected patients at baseline and EOT and in healthy subjects. They observed that the frequencies of the effector memory subsets of γδ T cells (CD45RA^-^CD27^-^) were reduced in HCV infected patients, while the number of naïve cells (CD45RA^+^CD27^+^) were increased, with no changes at EOT or 12 weeks post EOT compared to baseline. Patients who experienced relapse had lower frequencies of central memory cells (CD45RA^-^CD27^+^) and shifted towards the terminally differentiated effector subset (CD45RA^+^CD27^-^). Regarding Vδ2 T cells, DAA treatment reduced the higher frequencies of CD38^+^ cells seen in HCV infected patients compared with healthy subjects, while CD56 expression was found to be similar between the two groups. The CD56^+^ cytotoxic subset showed poor expansion potential upon stimulation which remained unchanged after DAA treatment (42).

MAIT cells are innate-like T cells, highly present in the human liver and mucosal lining of the gastrointestinal tract, with the majority expressing CD8 (43, 44). MAIT cells carry a semi-invariant TCR and are producers of cytokines like TNF-α, IFN-γ, IL-7, IL-12, and IL-17. Hengst et al. (44) conducted a study on 26 HCV infected patients and 12 healthy subjects to examine the global MAIT cell subset and the impact of DAA treatment. The study revealed a severe depletion of MAIT cells in the HCV-infected cohort compared to healthy subjects. Despite successful HCV clearance with DAA treatment, the MAIT cell frequency was not restored throughout the 48 weeks post-EOT. Additionally, phenotypical changes in MAIT cells were observed both before and after DAA treatment. Prior to treatment, MAIT cells expressed an activated phenotype characterised by high frequencies of granzyme B, HLA-DR, PD-1, and CD69 and lower expression of CD127 compared to healthy subjects. At EOT and 12 weeks post-EOT, expression of HLA-DR and CD69 partially normalised, while granzyme B and PD-1 levels remained elevated, and CD127 decreased. Upon stimulation, MAIT cells produced low levels of IFN-γ, TNF-α, and IL-17 in HCV infected patients compared to healthy subjects. No significant recovery was found in MR1-dependent MAIT cell responses post-DAA treatment. T-bet expression was slightly elevated in the HCV cohort and normalised after HCV clearance (44).

Lastly, Kochanowicz et al. (45) included 97 HCV infected patients in a study investigating double positive (DP) CD4^+^CD8^+^ T cells following successful DAA treatment. The function of DP T cells is not fully characterised, but they may play a role in adaptive immune responses and have been found in higher frequencies in the liver than in peripheral blood in HCV infected patients (45). Their study demonstrated that global DP T cells had higher PD-1 and lower Tim-3 expression than CD4^-^CD8^+^ T cells, with fewer PD-1^-^Tim-3^-^ cells than CD8^-^CD4^+^ T cells both before and after DAA treatment. PD-1^+^Tim-3^+^ DP T cells decreased post-treatment. HCV-specific DP T cells were more frequent and had lower PD-1, higher PD-1 and Tim-3 co-expression, and fewer PD-1^-^Tim-3^-^ cells compared to single-positive (SP) T cells (both CD4^+^ and CD8^+^) but maintained a distinct exhaustion phenotype after treatment (45).

Unconventional T cell subsets, including γδ T cells, MAIT cells, and DP T cells, also exhibit partial phenotypic and functional modulation after DAA treatment where signs of persistent activation or exhaustion remain, indicating incomplete immune restoration across these cell populations following viral clearance.

#### 3.4.4 Exhaustion of natural killer cells in chronic hepatitis C virus infection before and after DAA treatment

The human liver is highly enriched with NK cells, which have important roles in both innate and adaptive immunity. Like other types of lymphocytes, NK cells display signs of exhaustion and impairment with persistent antigen stimulation. These functional changes include increased cytotoxicity and decreased cytokine production (46, 47). However, in contrast to T cell exhaustion, which is more well defined, there is currently no clear consensus on the precise definition of NK cell exhaustion (46). NK cells also show downregulation of FcγRIII (CD16) with altered antibody-dependent cytotoxicity (ADCC) (47). NK cells can be subdivided into different subsets; here among immature NK^bright^ and mature NK^dim^. The mature NK^dim^ subset is defined as CD56^dim^ and is highly cytotoxic with abundant amounts of perforin and granzyme, while the immature NK^bright^ subset is defined as CD56^bright^ and is less cytotoxic but produces more cytokines (48).

Mele et al. (47) studied the effect of cure by DAA on the global NK cell subset in 59 HCV infected patients who were divided by fibrosis stage into group 1: fibrosis stage F3-F4 (≥9.5 kPa) and group 2: stage F1-F2 (<9.5 kPa) assessed by transient elastography compared to 34 healthy subjects. The study analysed the surface expression of PD-1, tumour necrosis factor-related-apoptosis-inducing ligand (TRAIL), and CD25 on NK cells, finding elevated levels of all these molecules compared with healthy subjects. Group 1 showed elevated levels of the activation marker CD69, and decreased levels of the inhibitory receptor Siglec-7 compared to group 2. The NK cell phenotypes in group 2 more closely resembled those of healthy subjects and did not change during the treatment period. During treatment, all receptors normalised, except for CD25, with an increase of Siglec-7 and a decrease of PD-1, TRAIL, and CD69. NKp46 MFI (mean fluorescence intensity) also decreased in group 2. Further, the authors investigated a recently discovered NK cell subset, adaptive NK cells (FcϵRIγ^neg^), which are identified by reduced expression of the intracellular gamma-signalling chain of the Fc receptor (FcϵRIγ) in mature CD57^+^ NK cells. Group 1 had an increased proportion of FcϵRIγ^neg^ cells, which decreased in both groups during treatment. Adaptive FcϵRIγ^neg^ cells had decreased expression of Siglec-7 and increased expression of PD-1 compared to FcϵRIγ^pos^ cells. PD-1 expression was the only molecule that decreased in group 1 during treatment. Additionally, IFN-γ production increased in both groups, while CD107a did not change in group 1 but increased in group 2. DAA treatment restored NK cell functional activity (antibody-dependent cell-mediated cytotoxicity (ADCC)) as measured by IFN-γ production by FcϵRIγ^neg^ cells (47).

Stevenson et al. (23) found a partial restoration of the NK cell subset. Immature NK cells decreased by 4 weeks post-treatment initiation, which was maintained at follow-up 12 weeks post EOT. No difference in the amount of mature NK cells was found after treatment at 12 weeks post EOT. From baseline to 12 weeks post EOT, the expression of natural toxicity receptors involved in the activation of NK cells, NKp30 and NKp46, as well as CD4 and Tim-3 expression, all decreased. TIGIT expression also decreased from baseline to EOT, but not at 12 weeks post-EOT (23). Szereday et al. (33) reported a decrease in the percentages of NK^bright^ cells from baseline to 12 and 24 weeks post-EOT. No difference was found in the frequency of NK and NK^dim^ cells. The expression of Tim-3 on NK^bright^ cells decreased during treatment. PD-1 was undetectable in NK cell subsets, but PD-L1 expression decreased in both NK and NK^dim^ cells (33).

The role of NK cells exhaustion after DAA treatment is less well characterised. However, the three included studies indicate that although DAA treatment leads to some restoration of NK cell phenotype and function, with reduction of expression of markers such as PD-1, TRAIL, and CD69 and enhanced IFN-γ production, some features of exhaustion persist, especially in patients with advanced fibrosis.

## 4 Discussion

T cell exhaustion is a state of dysfunction believed to be induced by persistent antigen stimulation during chronic viral infections, like chronic HCV infection, and cancer. T cell exhaustion is a progressive state induced by persistent antigen stimulation, characterised by impaired effector functions, limited T cell proliferation, reduced cytokine production, expression of co-inhibitory receptors, and epigenetic alterations (12, 13). Furthermore, the tolerogenic environment of the liver has immune-modulatory effect and plays a significant role in shaping immune responses during HCV-infection, with a pivotal role in HCV persistence (49). Previously, treatment that included pegylated interferon-α failed to restore T cell function after HCV cure (50, 51). Since the introduction of interferon-free DAA treatment in 2014, numerous studies have explored whether achieving cure can reinvigorate the dysfunctional immune response associated with chronic HCV infection. This systematic review summarises the current evidence on this topic. A simplified overview of the main findings is shown in **Table 2**.

**Table 2:** Changes in immune cell features after cure with DAA treatment in patients with chronic HCV infection.

Cell type	Frequency	Proliferation	Exhaustion markers	Cytokine production	Phenotype shift	Overall function
CD8+ T cells						
HCV-specific	↑	↑	↓	↑	↑	↑
Global	↔	↑/↔	↓	↔	↑	↔
CD4+ T cells						
HCV-specific	↑	↑	↓	↑	↑	↑
Global	↑	↑	↓	↑	↑	↑
Unconventional T cells						
γδ T cells	↔	↔	↓/↔		↔	↔
MAIT cells	↔		↔	↔	↔	↔
DP T cells	↔		↓		↔	
NK cells	↔		↓	↑	↑/↔	↑

Simplified table summarising the predominant changes in immune cells features after cure by DAA treatment found in the included studies. Immune features included frequency, proliferation, exhaustion markers, cytokine production, phenotype shift (e.g., to memory-like subsets), and overall function. An upwards arrow indicates relative increase, a downwards arrow indicates relative decrease, and a horizontal arrow indicates no change.

Broadly, the studies included in this review identified CD4^+^ and CD8^+^ T cells before DAA treatment to exhibit an exhausted phenotype, characterised by altered expression of activation markers, such as CD38, HLA-DR, ICOS, BTLA, and CD69 (27, 34), and by elevated EOMES and T-bet associated with a differentiated state of T cells and increased in exhaustion (27, 31, 38). Additionally, these T cells exhibited impaired cytokine and chemokine production with decreased levels of IFN-γ, TNF-α, MIP-1β, and interleukins, as well as heightened expression of molecules associated with terminal T cell exhaustion, including PD-1, CD39, TIGIT, CTLA-4 and Tim-3 (21, 26, 34, 39).

In most studies included in this review, DAA treatment partially restored central immune functions of CD8^+^ and CD4^+^ T cells. Several studies reported a decrease in PD-1 and/or Tim-3 expression on CD8^+^ T and/or CD4^+^ T cells, along with an increase in cytokine production like IL-2, TNF-α and IFN-γ following stimulation, after cure by DAAs (18, 23–25, 27, 28, 33, 35–37, 40). However, not all studies reported restoration of T-cell functions following DAA cure. Some studies found that several exhaustion parameters persisted post-DAA cure (22, 25, 31, 33, 39), and one study even observed an increase in some exhaustion markers after curative DAA treatment (24). These differences in findings could be attributed to the heterogeneity among the included study participants, including variations in age, sex, comorbidities, HCV genotype, HLA type, duration of infection, and fibrosis stage. Furthermore, the differences in methodological approaches likely account for the variability in the results. Some studies employ low-resolution techniques on global T cells, while others use advanced approaches providing a high-resolution view of HCV-specific responses, with the latter offering a more direct insight into antigen-driven exhaustion. When focusing specifically on HCV-specific CD8^+^ T cells after DAA treatment, some studies report partial improvement in the function of HCV-specific CD8^+^ T cells and reduction in exhaustion markers (18, 25). However, the majority of studies found that these cells continue to exhibit persistent exhaustion despite HCV clearance (19–22), indicating incomplete recovery. A challenge in interpreting the results is distinguishing between true reinvigoration of exhausted T cells and the selective survival of less-exhausted, memory-like subsets. For instance, CD8^+^ T cells possessing both memory potential and features of exhaustion were shown to persist after antigen clearance, while terminally exhausted subsets were lost, which aligns with findings in a murine LCMV model (52). Thus, the observed alterations in cell function and phenotype could partly reflect survival of an already existing cell subset rather than reversal of exhaustion per se, and this nuance is important when evaluating the effect of DAA treatment on exhaustion.

Several studies noted a link between the level of T cell exhaustion and the severity of fibrosis (24, 26, 28, 32, 38, 47). Patients with more severe fibrosis stage F3-F4 did not reinvigorate their immune functions as effectively as patients with milder fibrosis (stage F0-F1). This suggests that advanced liver damage prior to treatment may impair the ability of the immune system to fully recover, highlighting the importance of early intervention to prevent irreversible immune dysfunction. Fibrosis stage can be viewed as a proxy for the duration of infection, with more severe fibrosis typically indicating a longer period of infection. Therefore, the degree of fibrosis may not be the direct cause of persistent exhaustion, but rather, the extended length of time the individual has been exposed to the viral antigen. The studies included in this review generally do not provide information on duration of infection, making it difficult to fully assess this relationship.

Two studies reported a tendency towards persistent mitochondrial dysfunction and metabolic degranulation despite DAA cure (19, 29), indicating that cellular metabolism, crucial for T cell function, remains compromised even after viral clearance. These metabolic impairments could limit the energy supply necessary for effective immune responses, contributing to the sustained state of T cell exhaustion observed post-treatment. Some studies describe a shift in the phenotypes of CD8^+^ T cells from an exhausted state to a more memory-like type (20, 21, 27, 40), though still exhibiting some degree of exhaustion. This phenotypic shift may implicate a partial recovery where T cells begin to adopt characteristics associated with long-term immune memory, although not fully restored to a non-exhausted state. One study, with a long follow-up of four patients three years post-DAA treatment, still observed this same pattern (27), highlighting the chronic nature of the immune alterations caused by chronic HCV infection, despite cure after DAA treatment. Some studies concluded that the cells are left with a permanent “scar” even after the viral antigen is removed (19, 21, 22). Collectively, this review indicates that while DAA treatment is an effective cure for HCV, achieving full restoration of immune function may remain a challenge, particularly in patients with advanced fibrosis (often after prolonged infection) and persistent metabolic dysfunction in immune cells. This underscores the importance of timely intervention in achieving immune restoration, highlighting the necessity for early detection and prompt treatment of HCV infection.

Data on NK cell exhaustion before and after DAA treatment of chronic HCV infection is limited. The included studies found NK cells to be in an exhausted state with elevated levels of markers such as TRAIL, PD-1, and CD69 (47), which were all normalised post-treatment, as well as IFN-γ expression. A decrease in other activation markers, like NKp30, NKp46, and Tim-3, was also observed after DAA cure (47, 48), along with a phenotypic shift from the more cytotoxic NK^dim^ subset to the less cytotoxic NK^bright^ subset with higher cytokine production following stimulation (48).

Unconventional T cell subsets were also found to be exhausted during HCV infection. γδ T cells were found to be primarily within the effector memory subset but switched to a more naïve phenotype post-DAA cure. Still, even after cure, they continued to exhibit signs of exhaustion, such as elevated expression of activation marker CD56 on Vδ2 T cells and poor ability to expand after stimulation (42). The MAIT cell subset was found to be the most significantly reduced cell subset in HCV-infected individuals compared to healthy subjects, with high expression of activation markers, altered transcription factor expression profiles, and impaired cytokine production upon stimulation. This indicates that MAIT cells are phenotypically activated but functionally depressed during HCV infection, and the MAIT cell dysfunction seems to persist after successful HCV clearance, indicating that MAIT cell dysfunction may be non-reversible (44). The role of DP T cells is still unclear, but these cells appear to exhibit a distinct exhaustion phenotype even after treatment (45), making them an interesting T cell subset for further investigation. Overall, these findings suggest that various T cell subsets and NK cells display features of exhaustion. However, the molecular definition of exhaustion is most clearly established in CD8^+^ T cells. For other cell types, such as CD4^+^ T cells, MAIT cells, or NK cells, exhaustion may involve distinct or only partially overlapping mechanisms (13), which is important to consider in the interpretation of these observations.

Checkpoint inhibitors (CI) offer a potential therapeutic approach to restore immune function in conditions of immune exhaustion. CIs, including anti-PD1, anti-PD-1L, and anti-CTLA-4, are currently used for cancer immunotherapy and have demonstrated benefits in terms of survival and disease control in several advanced cancers (53). By blocking inhibitory pathways that contribute to T cell dysfunction, these agents can reinvigorate exhausted T cells, thereby restoring their ability to mount effective immune responses (54). Theoretically, this approach could also be exploited in chronic viral infections, such as HCV, where T cell exhaustion remains a barrier to full immune recovery, even after HCV clearance by DAAs. A systematic review investigated the safety and efficacy of CIs in cancer patients co-infected with HBV or HCV and concluded that these agents are generally safe (55). Therefore, further research into how CIs can be utilised in reversing the exhausted T cell phenotype in HCV infection is both interesting and warranted. However, the persistence of epigenetic scarring in T cells post-DAA cure (22) may limit the responsiveness to CIs and indicates that reversal of T cell exhaustion may require approaches beyond checkpoint blockade alone.

The development of an effective HCV vaccine has been underway for several decades, and some trial results in healthy populations have been promising (56–58). Unfortunately, results show less effectiveness in populations at higher risk of chronic HCV infection (8, 59). Given the WHO recent call to prioritise HCV vaccine research (9), advancing our understanding of T cell exhaustion across different T cell subsets is warranted to ultimately aid in vaccine development. Ensuring that a future vaccine will be effective for those who need it the most — particularly in vulnerable populations such as PWID and individuals with prior HCV infection and exhausted T-cells — is critical as these groups remain at high risk for re-infection (60). Additionally, it also remains to be explored how exhausted antigen-specific T cells behave during re-infection, as they may be less capable of challenging the newly infecting virus. Addressing T cell exhaustion could enhance the efficacy of vaccines and other therapeutic strategies, reducing the burden of HCV re-infection and improving health outcomes.

### 4.1 Strengths and limitations

To our knowledge, this is the first systematic review on T cell exhaustion before and after antiviral treatment with DAA in chronic HCV infection, which is a major strength of this study. However, a limitation is the lack of comprehensive data on the long-term effects on exhaustion across the different T cell subsets. Most studies have relatively short follow-up periods, with only a few studies extending beyond the immediate post-treatment phase, and just one study with a follow-up time point beyond one year (60 weeks) post-EOT. This limits our insights into prolonged changes in T cell phenotypes, and longer follow-up periods would be valuable to better understand the long-term changes, as immune recovery post-DAA treatment may require extended observation periods. Another limitation is the potential heterogeneity of the T cells across the populations. While some studies focused on HCV-specific T cells, others assessed global T cells responses, making it difficult to draw definite conclusions collectively across the studies. Global T cell responses may obscure those from HCV-specific T cells, introducing a potential confounder. Relatedly, the included studies vary considerably in methodological approaches and resolution, which influences the specificity and analytical depth of each study. Studies using advanced methods, such as HLA multimer sorting of HCV-specific CD8^+^ T cells followed by single-cell RNA sequencing, offer more detailed insights into antigen-driven T cell exhaustion than bulk phenotyping of global T cells. Of note, most included studies did not evaluate whether the analysed HCV-specific T cells were targeting circulating viral variants. Given the frequent viral escape mutations in chronic HCV infection, it is difficult to confirm whether ongoing TCR engagement was present. Additionally, not all studies adjusted for potential confounders such as age, sex, comorbidities, HCV genotype, duration of infection, fibrosis stage, and treatment regimens, complicating direct comparisons between studies. Finally, the omission of data on co-infections such as HIV and/or HBV in several studies introduces possible biases, as these infections can also impact exhaustion parameters.

### 4.2 Conclusion

Overall, the studies included in this systematic review demonstrate that curative DAA treatment leads to some degree of immune restoration in exhausted T cell populations, although the extent of this restoration varies. Prior to DAA treatment, T cells showed exhaustion-linked alterations in their phenotype, characterised by upregulation of inhibitory receptors, reduced cytokine production, and impaired proliferative potential. While some studies observed normalisation of several exhaustion markers post-DAA treatment, others reported “scarring” of the T cell phenotype with several persisting exhaustion markers. Whether this apparent partial functional recovery reflects true reversal of exhaustion or if it partly reflects selective survival of less exhausted, memory-like T cell subsets remains unclear. The extent of the “scarring” seems to be dependent on the duration of infection as indicated by higher degrees of exhaustion with higher degrees of fibrosis. This indicates that the continuous antigen stimulation during chronic HCV infection may imprint irreversible changes on the T cells. However, the long-term impact on T cell populations remains insufficiently characterised, as only a limited number of studies investigated this aspect.

Further research is needed to comprehensively understand the prolonged effects of DAA treatment on T cell exhaustion dynamics and to understand how exhaustion can be reversed following DAA cure. Lastly, exploring potential confounders such as age, sex, HCV genotype, duration of HCV infection, and fibrosis stage may also be important for understanding the barriers to immune restoration and for informing the future development of a vaccine.

## Data Availability

The original contributions presented in the study are included in the article/**Supplementary Material**. Further inquiries can be directed to the corresponding author.

## Glossary

γδ T cell: Gamma-delta T cells
4-1BB/4-1BBL: Receptor ligand for the immune checkpoint TNF
ADCC: Antibody-dependent cell-mediated cytotoxicity
BTLA: B and T lymphocyte attenuator
CD: Cluster of differentiation
ChARs: Chromatin-accessible regions
CTLA-4: Cytotoxic T-lymphocyte associated protein 4
DAAs: Direct Acting Antivirals
DP: Double positive
Endocab IgG: Endotoxin-core IgG antibody
Eomes: Eomesodermin
EOT: End Of Treatment with DAAs
HCV: Hepatitis C virus
HIV: Human immunodeficiency virus
HLA-DR: Humane leukocyte antigen DR
HIF-1α: Hypoxia-inducible factor 1-α
ICOS: Inducible co-stimulator
IFN: Interferon
IL: Interleukin
IP10: IFN-gamma-inducible protein 10
LAG-3: Lymphocyte-activation gene 3
LBP: Lipopolysaccharide-binding protein
MAIT: Mucosal-associated invariant T cells
MIP-1β: Macrophage inflammatory protein-1beta
NFAT: Nuclear factor of activated T cells
NK cells: Natural killer cells
PD-1: Programmed cell death-1
PWID: Persons who inject drugs
SP: Single-positive
SVR: Sustained Virological Response
TCF-1: T cell factor-1
TCR: T cell receptor
T_ex_: Exhausted CD8^+^ T cell
Tfh cells: T follicular helper cells
TIGIT: T cell immunoreceptor with Ig and ITIM domains
Tim-3: T cell immunoglobulin and mucin-domain containing-3
TNF: Tumour necrosis factor
TOX: Thymocyte selection-associated high mobility group box protein
TRAF1: The signal transducer of the immune checkpoint receptor ligand 4-1BB/4-1BBL
TRAIL: Tumour necrosis factor-related-apoptosis-inducing ligand
ZAP-70: Zeta-chain-associated protein kinase 70
