# Supplementary material for: Cure of chronic hepatitis C virus infection after DAA treatment only partially restores the functional capacity of exhausted T cell subsets: a systematic review
Source: Front Immunol. 2025 Sep 1;16:1546915. doi: 10.3389/fimmu.2025.1546915 (PMC12434116; doi:10.3389/fimmu.2025.1546915)
Supplement: Supplementary file 1 [file DataSheet1.docx]

Supplementary Material

**Cure of chronic hepatitis C virus infection after DAA treatment only partially restores the functional capacity of exhausted T cell subsets: a systematic review**

**Ása Didriksen Apol^1,2^, Christina Sølund^1^, Caroline Vinten^1^, Alexander P. Underwood^1,2^, Jens Bukh^1,2^, Nina Weis^1,3*^.**

^1^ Department of Infectious Diseases, Copenhagen University Hospital, Hvidovre, Hvidovre, Denmark

^2^ Copenhagen Hepatitis C Program (CO-HEP), Department of Infectious Diseases, Copenhagen University Hospital Hvidovre and Department of Immunology and Microbiology, Faculty of Health and Medical Sciences, University of Copenhagen, Copenhagen, Denmark

^3^Faculty of Health and Medical Sciences, Department of Clinical Medicine, University of Copenhagen, Copenhagen, Denmark.

**Table of content**

Supplementary table 1...………………………………………………………………………...……3

Search string Pubmed………………………………………………………………………….……..4

Search string Embase…………………………………………………………………………...…….5

| **Supplementary table 1. Search string.** | | | | | | | |
| --- | --- | --- | --- | --- | --- | --- | --- |
| **Keywords** | **Aspect 1:**  T cell |  | **Aspect 2:**  Hepatitis C |  | **Aspect 3:**  Direct acting antivirals |  | **Aspect 4:**  Exhaustion |
| **MeSH** | T-lymphocytes | **A**  **N**  **D** | Hepatitis C | **A**  **N**  **D** | Antiviral agents | **A**  **N**  **D** |  |
| **Free text** | "t lymphocyte*" OR "t-lymphocyte*" OR "t cell*" OR "t-cell*" OR "cd8 cell*" OR “cd8-cell*” OR "cd8 t cell*" OR “cd8 t-cell*”OR “cd8 lymphocyte*” OR “cd8-lymphocyte*” OR "cd8 positive" OR “cd8-positive” OR  "cd4 cell*" OR “cd4-cell*” OR "cd4 t cell*" OR “cd4 t-cell*”OR “cd4 lymphocyte*” OR “cd4-lymphocyte*” OR “cd4+ lymphocyte*” OR “cd4+-lymphocyte*” OR "cd4 positive" OR “cd4-positive” OR  "memory cell*" OR “memory-cell*” OR "memory t cell*" OR “memory t-cell*”OR “memory lymphocyte*” OR “memory-lymphocyte*” OR  "cytotoxic cell*" OR “cytotoxic-cell*” OR "cytotoxic t cell*" OR “cytotoxic t-cell*”OR “cytotoxic lymphocyte*” OR “cytotoxic-lymphocyte*” OR  "nk cell*" OR “nk-cell*” OR “natural killer cell*” OR “natural-killer cell*” OR “natural killer t cell*” OR “natural killer t-cells*” OR “natural killer lymphocyte*” OR “natural-killer t cell*” OR “natural-killer t-cells*” OR “natural-killer lymphocyte*” OR  "regulatory cell*" OR “regulatory-cell*” OR "regulatory t cell*" OR “regulatory t-cell*”OR “regulatory lymphocyte*” OR “regulatory-lymphocyte*” OR “tex” OR “tex cell*” OR “tex-cell*” | **A**  **N**  **D** | ”hepatitis c” OR ”hep c” OR HCV OR “hepacivirus c” | **A**  **N**  **D** | ”direct acting antiviral*” OR “antiviral*” OR “antiviral agent*” OR “antiviral drug*” or “antiviral therapy*” OR “DAA” | **A**  **N**  **D** | exhaustion OR exhausted OR dysfunction |

**Search string Pubmed**

((("T-Lymphocytes"[Mesh]) OR ("t lymphocyte*"[Text Word] OR "t-lymphocyte*"[Text Word] OR "t cell*"[Text Word] OR "t-cell*"[Text Word] OR "cd8 cell*"[Text Word] OR "cd8-cell*"[Text Word] OR "cd8 t cell*"[Text Word] OR "cd8 t-cell*" OR "cd8 lymphocyte*"[Text Word] OR "cd8-lymphocyte*"[Text Word] OR "cd8+ lymphocyte*"[Text Word] OR "cd8+-lymphocyte*"[Text Word] OR "cd8 positive"[Text Word] OR "cd8-positive"[Text Word] OR "cd4 cell*"[Text Word] OR "cd4-cell*"[Text Word] OR "cd4 t cell*"[Text Word] OR "cd4 t-cell*" OR "cd4 lymphocyte*"[Text Word] OR "cd4-lymphocyte*"[Text Word] OR "cd4+ lymphocyte*"[Text Word] OR "cd4+-lymphocyte*"[Text Word] OR "cd4 positive"[Text Word] OR "cd4-positive"[Text Word] OR "memory cell*"[Text Word] OR "memory-cell*" OR "memory t cell*"[Text Word] OR "memory t-cell*" OR "memory lymphocyte*" OR "memory-lymphocyte*" OR "cytotoxic cell*" OR "cytotoxic-cell*" OR "cytotoxic t cell*" OR "cytotoxic t-cell*" OR "cytotoxic lymphocyte*" OR "cytotoxic-lymphocyte*" OR "nk cell*" OR "nk-cell*" OR "natural killer cell*" OR "natural-killer cell*" OR "natural killer t cell*" OR "natural killer t-cells*" OR "natural killer lymphocyte*" OR "natural-killer t cell*" OR "natural-killer t-cells*" OR "natural-killer lymphocyte*" OR "regulatory cell*" OR "regulatory-cell*" OR "regulatory t cell*" OR "regulatory t-cell*" OR "regulatory lymphocyte*" OR "regulatory-lymphocyte*" OR "tex"[Text Word] OR "tex cell*" OR "tex-cell*")) AND (("Hepatitis C"[Mesh]) OR ("hepatitis c"[Text Word] OR "hep c"[Text Word] OR HCV[Text Word] OR "hepacivirus c"[Text Word])) AND (("Antiviral Agents"[Mesh]) OR ("direct acting antiviral*" OR "antiviral*" OR "antiviral agent*" OR "antiviral drug*" OR "antiviral therapy*" OR "DAA")) AND (exhaustion[Text Word] OR exhausted[Text Word] OR dysfunction[Text Word]))

**Search string Embase**

((T-lymphocyte.mp.)

OR

(t lymphocyte*.tw. OR t-lymphocyte*.tw. OR t cell*.tw. OR t-cell*.tw. OR cd8 cell*.tw. OR cd8-cell*.tw. OR cd8 t cell*.tw. OR cd8 t-cell*.tw. OR cd8 lymphocyte*.tw. OR cd8-lymphocyte*.tw. OR cd8+ lymphocyte*.tw. OR cd8+-lymphocyte*.tw. OR cd8 positive.tw. OR cd8-positive.tw. OR cd4 cell*.tw. OR cd4-cell*.tw. OR cd4 t cell*.tw. OR cd4 t-cell*.tw. OR cd4 lymphocyte*.tw. OR cd4-lymphocyte*.tw. OR cd4+ lymphocyte*.tw. OR cd4+-lymphocyte*.tw. OR cd4 positive.tw. OR cd4-positive.tw. OR memory cell*.tw. OR memory-cell*.tw. OR memory t cell*.tw. OR memory t-cell*.tw. OR memory lymphocyte*.tw. OR memory-lymphocyte*.tw. OR cytotoxic cell*.tw. OR cytotoxic-cell*.tw. OR cytotoxic t cell*.tw. OR cytotoxic t-cell*.tw. OR cytotoxic lymphocyte*.tw. OR cytotoxic-lymphocyte*.tw. OR nk cell*.tw. OR nk-cell*.tw. OR natural killer cell*.tw. OR natural-killer cell*.tw. OR natural killer t cell*.tw. OR natural killer t-cells*.tw. OR natural killer lymphocyte*.tw. OR natural-killer t cell*.tw. OR natural-killer t-cells*.tw. OR natural-killer lymphocyte*.tw. OR regulatory cell*.tw. OR regulatory-cell*.tw. OR regulatory t cell*.tw. OR regulatory t-cell*.tw. OR regulatory lymphocyte*.tw. OR regulatory-lymphocyte*.tw. OR tex.tw. OR tex cell*.tw. OR tex-cell*.tw.))

AND

((Hepatitis C.mp.)

OR

(hepatitis c.tw. OR hep c.tw. OR HCV.tw. OR hepacivirus c.tw.))

AND

((Antiviral agents.mp.)

OR

(direct acting antiviral*.tw. OR antiviral*.tw. OR antiviral agent*.tw. OR antiviral drug*.tw. OR antiviral therapy*.tw. OR DAA.tw.))

AND

(exhaustion.tw. OR exhausted.tw. OR dysfunction.tw.)
